# Insecticide resistance selection and reversal in two strains of *Aedes aegypti*

**DOI:** 10.12688/wellcomeopenres.15974.2

**Published:** 2020-12-16

**Authors:** Jonathan Thornton, Bruno Gomes, Constância Ayres, Lisa Reimer

**Affiliations:** 1 Vector Biology Department, Liverpool School of Tropical Medicine, Liverpool, L35QA, UK; 2 Laboratório de Bioquímica e Fisiologia de Insetos, Oswaldo Cruz Institute (IOC-FIOCRUZ), Rio de Janeiro, 21040-360, Brazil; 3 Aggeu Magalhães Institute, Oswaldo Cruz Foundation (IAM-FIOCRUZ), Recife, Brazil

**Keywords:** Aedes aegypti, insecticide resistance, mosquito, pyrethroid, permethrin, temephos, malathion, kdr

## Abstract

**Background**: Laboratory reared mosquito colonies are essential tools to understand insecticide action. However, they differ considerably from wild populations and from each other depending on their origin and rearing conditions, which makes studying the effects of specific resistance mechanisms difficult. This paper describes our methods for establishing multiple resistant strains of *Aedes aegypti* from two colonies as a new resource for further research on metabolic and target site resistance.

**Methods**: Two resistant colonies of *Ae. aegypti*, from Cayman and Recife, were selected through 10 generations of exposure to insecticides including permethrin, malathion and temephos, to yield eight strains with different profiles of resistance due to either target site or metabolic resistance. Resistance ratios for each insecticide were calculated for the selected and unselected strains. The frequency of *kdr* alleles (F1534C and V1016I) in the Cayman strains was determined using TaqMan assays. A comparative gene expression analysis among Recife strains was conducted using qPCR in larvae (CCae3A, CYP6N12, CYP6F3, CYP9M9) and adults (CCae3A, CYP6N12, CYP6BB2, CYP9J28a).

**Results**: In the selected strain of Cayman, mortality against permethrin reduced almost to 0% and *kdr* became fixated by 5 generations. A similar phenotype was seen in the unselected homozygous resistant colony, whilst mortality in the susceptible homozygous colony rose to 82.9%. The Recife strains showed different responses between exposure to adulticide and larvicide, with detoxification genes in the temephos selected strain staying similar to the baseline, but a reduction in detoxification genes displayed in the other strains.

**Conclusions**: These selected strains, with a range of insecticide resistance phenotypes and genotypes, will support further research on the effects of target-site and/or metabolic resistance mechanisms on various life-history traits, behaviours and vector competence of this important arbovirus vector.

## Introduction

*Aedes aegypti* is one of the most significant mosquito species of public health concern due to its role as a vector of several arboviruses, including dengue, yellow fever, chikungunya and Zika. Over 4 million disability-adjusted life years worldwide were attributed to mosquito-borne viruses in 2013 ( Moyes *et al.*, 2017). Dengue virus, the most ubiquitous arbovirus, is found in 128 countries across temperate and tropical regions, and 3.9 billion people currently live at risk of infection ( Guzman & Harris, 2015; Pollett *et al.*, 2018). Yellow fever has re-emerged as an important disease in Africa and the Americas, with outbreaks occurring in regions that previously had low vaccination coverage and low-to-zero yellow fever incidence ( Douam & Ploss, 2018; Monath & Vasconcelos, 2015). Large outbreaks of chikungunya virus have also been described since 2000 ( Burt *et al.*, 2017). Meanwhile, the World Health Organization declared a “public health emergency of international concern” during the Zika virus epidemic of 2015 and following the discovery of its association with microcephaly ( Kindhauser *et al.*, 2016).

Vector control is the primary strategy to prevent transmission of arboviruses due to the absence of prophylactic drugs or vaccines for most diseases ( Silva *et al.*, 2018). Chemical insecticides, biological agents, and habitat management ( George *et al.*, 2015; Horstick *et al.*, 2010) are three common methods of controlling *Aedes spp.* However, insecticide resistant *Ae. aegypti* are commonly reported in Latin America and southern Asia, and have been reported in Africa ( Moyes *et al.*, 2017; Vontas *et al.*, 2012), threatening the efficacy of vector-borne disease control programs ( Corbel *et al.*, 2017; Moyes *et al.*, 2017; Ranson *et al.*, 2010).

Experimental studies comparing the attributes of susceptible and resistant mosquito colonies are crucial to elucidate resistance mechanisms ( Davies *et al.,* 2008; Feyereisen *et al.*, 2015; Ingham *et al.,* 2018; Li *et al.,* 2007; Melo-Santos *et al.*, 2010; Moyes *et al.,* 2017; Poupardin *et al.*, 2014; Ranson *et al.*, 2010; Rinkevich *et al.,* 2013; Stevenson *et al.*, 2012; Strode *et al.*, 2012; Vontas *et al.*, 2012; Weetman *et al.*, 2018), insecticide mode of action ( Ffrench-Constant, 2013; Yunta *et al.*, 2016), and the fitness costs of resistance ( Brito *et al.*, 2013; Diniz *et al.*, 2015). This information is necessary to develop new insecticide formulations and alternative control methods that avoid cross-resistance ( de Lourdes Macoris *et al.*, 2018; Jankowska *et al.*, 2017). However, differences between susceptible and resistant colonies due to differing genetic backgrounds – caused by bottlenecks or genetic drift – may influence study outcomes ( Ffrench-Constant, 2013). The ability to select sub-strains from a single parent colony which exhibit phenotypically distinct insecticide susceptibility profiles could help address the limitations of using disparate colonies. The aim of our study was to select multiple strains of *Ae. aegypti*, through exposure to a range of insecticides, that vary in resistance phenotype. Here we present the resistant phenotype after ten generations of insecticide exposure using two parent colonies: CAYMAN, a pyrethroid-resistant colony conferred by two target-site mutations in the sodium channel gene (V1016I and F1534C) ( Harris *et al.*, 2010), and RECIFE, a temephos-resistant colony conferred by overexpression of multiple detoxification genes ( Diniz *et al.*, 2015). Additionally, we present differences in a select number of resistant alleles and metabolic genes which can be used to inform further research.

## Methods

### Summary of the study design

A Total of three colonies from Aedes aegypti were used in this study:

Colony CAYMAN (CAY) was originally established in 2008 with Aedes aegypti collected in Grand Cayman island (Caribbean). This colony is highly resistant to pyrethroids and DDT, attributed to kdr alleles (F1534C and V1016I), and has been routinely selected with 0.75 % permethrin for 1 hour in the Liverpool Insect Testing Establishment (LITE: https://lite.lstmed.ac.uk/lite-facilities/lite-insectaries/aedes-aegypti-cayman).

Colony RECIFE (REC) was originally established in 2004 with Aedes aegypti collected in Araripina, Brazil (7° 32’ S and 40° 34’ W; Melo-Santos *et al*., 2010). This colony is resistant to temephos (OP), and biochemical assays indicate a higher activity of multiple detox enzyme families. Larvae from this colony have been routinely selected with 0.5 mg/L temephos for 24 hours.

Colony NEW ORLEANS is a susceptible colony established at LSTM in the 1970s. This colony originated in New Orleans (USA) and it is maintained in laboratory without insecticide exposure. Routine screening for target-site mutations associated with resistance indicates a lack of kdr alleles in the sodium channel and resistant alleles in Ace-1 (LITE: https://lite.lstmed.ac.uk/lite-facilities/lite-insectaries/aedes-aegypti-new-orleans).

**Figure 1.**
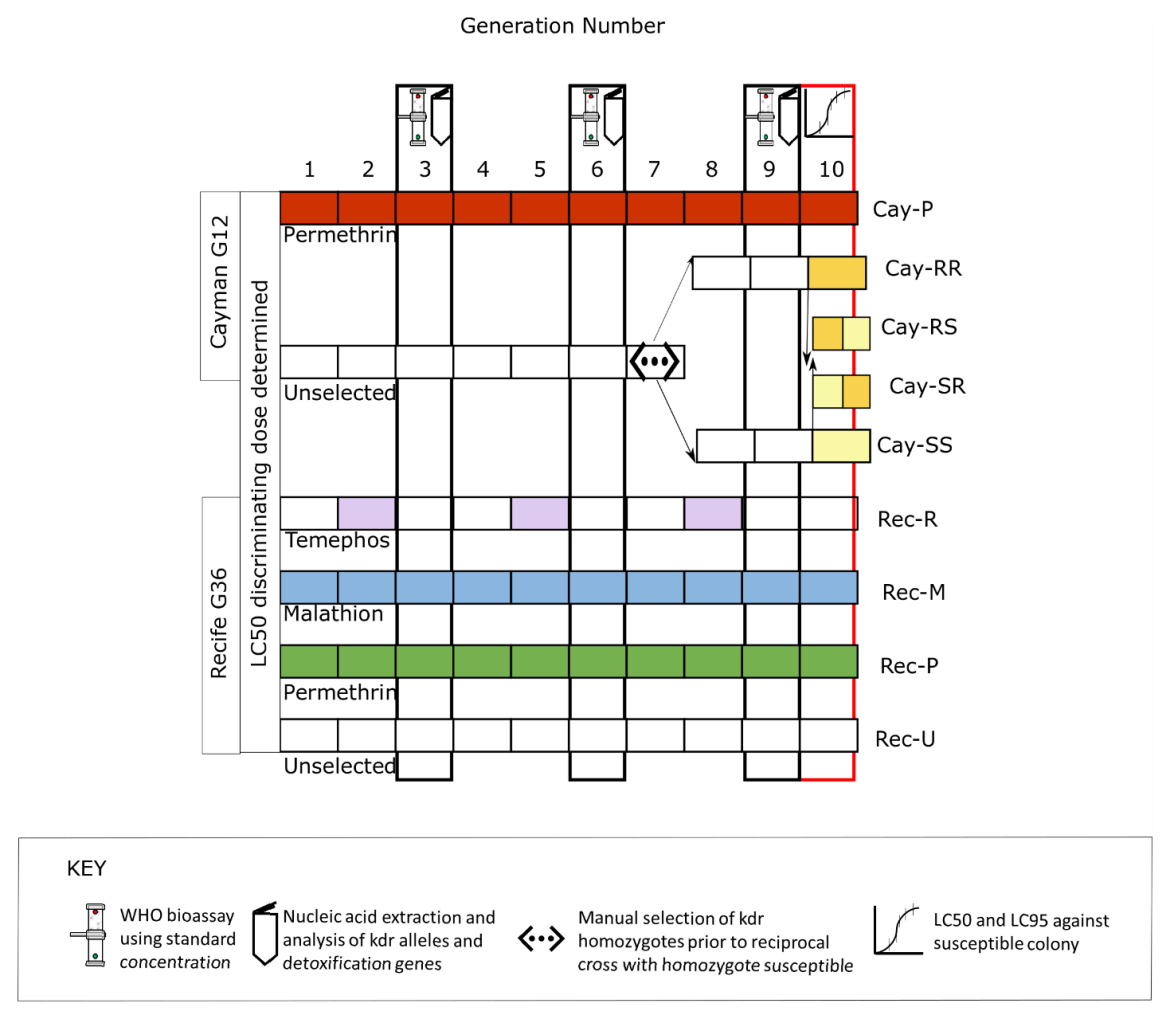
Experiment outline. Strains used were Cayman, which has target-site resistance to pyrethroids/DDT, and Recife, which has metabolic resistance to temephos. LC50s were determined by discriminating dose assays, then the LC50 was used to select the strain. Cayman was split into a strain selected with permethrin and an unselected strain, which was manually selected into R and S homozygotes and then crossed with reciprocal homozygotes to produce heterozygote strains. Recife was selected with malathion and permethrin, one strain was maintained with temephos exposure every three generations and one strain was left unselected. Each strain was subjected to bioassays using the WHO tube assay at the WHO recommended concentrations every three generations, and nucleic acid extraction and analysis of *kdr* alleles or detoxification genes was performed. At the end of 10 generations, each strain underwent bioassays to determine the LC50 and LC95 compared to the susceptible strain.

We exposed mosquitoes from CAY and REC colonies to different insecticide selection pressures and monitored key indicators of resistance over time ( Figure 1). We established the following eight strains of *Ae. aegypti*: CAY-P exposed to permethrin, CAY-RR unexposed and homozygous for resistance alleles (V1016I and F1534C), CAY-RS unexposed and heterozygous for resistance alleles, CAY-SS unexposed and homozygous for susceptible alleles, REC-R exposed to temephos, REC-M exposed to malathion, REC-P exposed to permethrin, and REC-U unexposed.

First, discriminating concentration assays were conducted to identify a suitable concentration of permethrin, malathion and temephos for the selection procedure. Second, mosquitoes were exposed to an insecticide selection regime as outlined in Figure 1. Every three generations during the selection process, we monitored phenotypic resistance according to WHO diagnostic concentrations and we assessed the presence of *kdr* alleles (CAY) or the upregulation of a select panel of detox genes (REC). Finally, resistance ratios for the eight strains were compared against the fully susceptible colony (NO). Details of these procedures are described below.

### Initial Discriminating Concentration assays in larvae and adults

Discriminating concentration assays using WHO tubes were conducted to determine an appropriate concentration of insecticide to expose each colony. The criteria is based on LC _50_ to provide insecticide pressure, but within concentrations that mosquitoes are likely to encounter in the field. Papers impregnated with insecticide were prepared according to the standard WHO protocol ( WHO, 2013). Filter papers of 12 cm × 15 cm were impregnated with a 1:1:1 volume mix of insecticide, acetone and corning oil and left to dry in a fume hood for 24 hours. Papers were stored wrapped in aluminium foil and placed in plastic bags at -20°C and used in up to five assays or within six months. The adult mosquitoes were selected with either permethrin (Sigma Aldritch, Pestanal®, CAS # 52645-53-1, >95.0% sum of cis+trans 97.8%) or malathion (Sigma Aldritch, Pestanal®, CAS # 121-75-5) and larvae were selected with temephos (CHEM SERVICE INC., CAS # 3383-96-8) (see “ *Mosquito selection regime*”). Groups of 25 L3 larvae were exposed to different concentrations of temephos in 200 mL of water to confirm the current LC _50_. The LC50 identified for each colony-insecticide combination was used in subsequent selection procedures described below. However, we could not achieve an LC50 in the CAY/permethrin combination that was relevant to concentrations that mosquitoes would typically encounter. We therefore decided to expose CAY to a high concentration (3%) that is similar to doses received in the wild.

### Mosquito selection regime

*Cayman colony*. We exposed 2–5 day old adult female mosquitoes in one strain, CAY-P, to 3% permethrin every generation according to the standard WHO protocol ( WHO, 2013). After a one-hour exposure and 24 hours recovery time, mortality was recorded and the surviving adults were allowed to mate and bloodfeed to create the next generation. We maintained another strain without insecticide exposure for ten generations (CAY-U). After five generations without insecticide exposure (G1–G6), we used molecular tools to manually split CAY-U (see “Manual selection of *kdr* alleles”) in generations 6 and 8 due to the high frequency of *kdr* alleles (I1016 and C1534). Four unselected strains were established: i) homozygous susceptible individuals (CAY-SS: V1016 and F1534), ii) homozygous resistant individuals (CAY-RR *:* I1016 and C1534), and iii) two heterozygote strains created by crossing resistant females with susceptible males (CAY-RS) or susceptible females with resistant males (CAY-SR) (see Figure 1).

*Recife colony.* We selected larvae with temephos every three generations to create REC-R ( Melo-Santos *et al.*, 2010). Groups of L3 larvae were exposed to 0.5 mg/L temephos in plastic trays for 24 hours. At 24 hours, the mortality was recorded and surviving larvae were transferred to fresh water, provided with food, and allowed to pupate and emerge as usual. Adult females were allowed to mate and bloodfeed to create the next generation. Two additional strains were established by exposing 2–5 day old adult female mosquitoes to malathion (REC-M: 1% WHO papers for 6 generations and 1.5% WHO papers for 3 generations) or permethrin (REC-P: 0.4% WHO papers for 6 generations and 0.75% WHO papers for 3 generations) every generation according to the standard WHO protocol ( WHO, 2013) (see Figure 1). The exposure concentrations were determined by the initial discriminating dose. Survivors were allowed to mate and bloodfeed to create the next generation. The concentration was increased at G7 following a decrease in mortality to 25% after exposure in G6 (see *Extended data:* Table S1 ( Reimer, 2020)).

### Evaluating phenotypic resistance

We performed WHO tube assays ( WHO, 2013) pre-selection and every three generations (G3, G6 and G9) during the selection regime for permethrin or malathion. Assays were performed with standard WHO papers at diagnostic concentration (0.75% for permethrin, 5% for malathion), ordered from the Vector Control Research Unit, School of Biological Sciences, Universiti Sains Malaysia. Four exposure tubes and one negative control tube were filled with up to 25 mosquitoes each. Mosquitoes were exposed for one hour, returned to the holding tubes and provided with 10% sucrose solution. Mortality was recorded after 24 hours.

### Detection of kdr alleles

For DNA analysis, 50 female mosquitoes were analysed every third generation of selection. Mosquitoes were killed at -20°C and stored on silica gel. We extracted DNA from individual mosquitoes using the Livak method ( Livak, 1984). TaqMan® SNP Genotyping Assays for *Vgsc-*1016 and *Vgsc*-1534 alleles in *Aedes* ( *Extended data:* Table S2 ( Reimer, 2020)) were used to screen for *kdr* in the Cayman strains. The lack of resistant *kdr* alleles at *Vgsc-*1016 and *Vgsc*-1534 was confirmed in a small subsample of Recife pre-selection (G17). TaqMan reactions were performed in 10 μl volumes containing 1X TaqMan® Gene Expression Master Mix (Thermo Fisher Scientific, MA, USA), 800 nM of each primer, and 200 nM of each probe on an Mx3005P qPCR thermal cycler (Agilent Technologies, CA, USA) with initial denaturation of 10 min at 95°C followed by 40 cycles of 15 s at 92°C and 1 min at 60°C.

### Detoxification gene expression

For RNA analysis, total RNA was extracted from pools of five female mosquitoes using Quick-RNA™ Miniprep (Zymo Research, CA, USA) and the purity and quantity of RNA were individually determined using a Nanodrop spectrophotometer (Nanodrop Technologies, DE, USA). SuperScript® III First-Strand Synthesis System performed cDNA synthesis from total RNA using oligo-dT20 primer (Thermo Fisher Scientific). Four genes associated with insecticide resistance were selected to screen expression profiles in larvae (CCae3A, CYP6N12, CYP6F3 and CYP9M9: all associated with temephos resistance) and adults (CCae3A and CYP6N12, associated with temephos resistance; and CYP6BB2 and CYP9J28a, associated with pyrethroid resistance; Moyes *et al*., 2017). cDNA was diluted ten-fold and qPCR reactions were performed in 20 μl volumes containing 2 μl of cDNA, 1x PowerUp™ SYBR® Green Master Mix (Thermo Fisher Scientific) and 300 nM of each primer on an Mx3005P qPCR thermal cycler (Agilent Technologies) with initial denaturation of 10 min at 95°C followed by 40 cycles of 15 s at 92°C and 30 s at specific TA ( *Extended data:* Table S3 ( Reimer, 2020)). The specificity of the primers was verified by melting curve analyses.

Relative fold gene expression was calculated using the comparative CT method (2 ^-ΔΔCt^ method), taking into account PCR efficiency ( Pfaffl, 2001). The genes coding for the 60S ribosomal protein L8 (RPL8) and the 40S ribosomal protein S7 (RPS7) were defined as reference genes. Between three and five biological replicates were performed for each strain and REC as baseline, respectively. All samples were run in duplicate. Results were expressed as mean transcription ratio in each strain and life stage ± SD relative to the mean transcription ratio of the specific life-stage of REC. Mann–Whitney U tests from the R package “stats” (R version 4.01; Copyright (C) 2020 The R Foundation for Statistical Computing) were used to compare transcription ratios between the selected strains and REC. The sequential Holm-Bonferroni procedure ( Holm, 1979) was used to adjust *α* to account for multiple comparisons.

### Manual selection of kdr alleles

CAY-U was maintained without insecticide pressure for five generations (G1–G6) before starting the selection process to establish homozygous strains for *Vgsc*-1016 and *Vgsc*-1534, which over the course of the process showed complete linkage in all individuals. These strains were established by separating pupae by sex and removing a leg from each adult for genotyping, as described above, before returning the adults to a cage for mating. The process was divided into two steps: 1) in generation G6, all the isoleucine homozygote mosquitoes for *Vgsc*-1016 were removed, and only heterozygotes and valine homozygotes were allowed to mate; 2) in generation G8, two homozygous strains were established for *Vgsc*-1534 (CAY-SS: Phe/Phe; CAY-RR: Cys/Cys), and all the heterozygotes were removed. Both CAY-SS and CAY-RR were screened by TaqMan assays for *Vgsc*-1016 and *Vgsc*-1534 in generation G9 to confirm the genotype of each strain. An extra selection was repeated in G9 for CAY-SS to remove the few individuals with *kdr* alleles.

### Determination of resistance ratio based on LC _50_ and LC _95_

Standardised larval trays were prepared with 200 L1 larvae and provided a yeast tablet every other day until pupation. Adult two to five day old female mosquitoes were exposed to insecticide papers of a range of concentrations of malathion and permethrin, as described previously. Mortality was calculated 24 hours after exposure, and at least three replicates of each assay were performed. For larval bioassays, stock concentrations of temephos were prepared at 0.05 – 1 mg/ml. 1 ml of stock was added to 750 ml of water mixed in a 1:1 ratio from distilled water and larval water from the trays. The water was mixed and aliquoted into five pupae pots of 150 ml each and groups of 25 L3 larvae in 50 ml water were added to each. This process was repeated for each concentration in the assay. Larval mortality was recorded at 24 hours and Abbot’s formula ( Abbott, 1925) was used to adjust mortality when necessary. The data from bioassays was organized by concentration in each biological replicate. Thisese data waswere used to create Ggeneral linear models (G.L.M.) with “logit” models for each strain using “glm” function in statistical software R-2.15.2. Lethal concentration and their95% confidenceant intervals at 95% were calculated using the r-package “MASS”. Resistance ratios were calculated based on comparison of LC50 and LC95 from each strain against the reference colony New Orleans, which is fully susceptible to all three insecticides. Confidence intervals for ratios were calculated using the method MOVER-R ( Newcombe, 2016) presented in the R-package “pairwiseCI”.

### Mosquito colony maintenance

All mosquitoes were maintained in the insectary of the Liverpool School of Tropical Medicine under controlled temperature (26 ± 2°C), relative humidity (75 ± 20%), and photoperiod (12:12 L:D). Adult mosquitoes were housed in BugDorm cages (MegaView Science Co, Ltd, Taiwan) and provided with constant access to 10% sucrose solution on a cotton pad, which was changed weekly. Eggs were obtained by feeding mated adult females on blood using a Hemotek feeder (Hemotek Ltd, Blackburn, UK). Due to issues with our supplier, the blood source was changed from human to horse at the beginning of the selection procedures. However, issues with mosquito egg-laying performance forced us to switch back to human blood mixed from separate bags of red blood cells and plasma in a 50:50 ratio from supplier overstock. Larvae were reared in plastic trays and fed Brewer’s Yeast tablets (Nature’s Aid ®).

## Results

### Cayman

**Figure 2.**
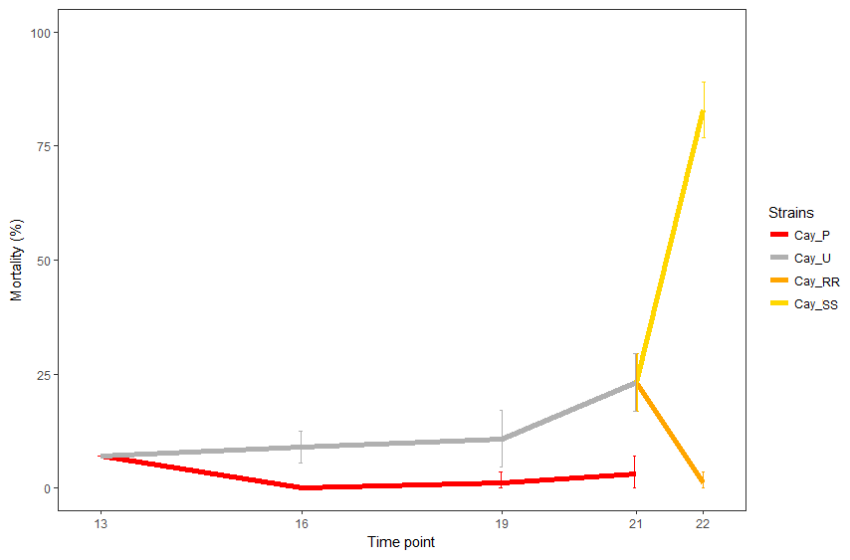
Mortality of Cayman strains against WHO standard permethrin 0.75%. The mortality profile of the CAYMAN strain continuously exposed to permethrin (CAY-P) is compared to the unexposed strain after exposure to 0.75% permethrin in the WHO bioassay. By generation 21, CAY-U had been split into *kdr* homozygous individuals (CAY-RR) and homozygous susceptible (CAY-SS) and the bioassay was repeated in generation 22. Bars represent 95% confidence intervals.

In the unexposed strain CAY-U, we observed a slight increase in mortality over time for the standard WHO bioassay (0.75% permethrin) ( Figure 2). In the permethrin selected CAY-P strain, mortality decreased to nearly 0% compared to the baseline of 4.6% mortality after only three generations of insecticide exposure at 3% permethrin. The *kdr* resistant alleles were still present in CAY-U at a high frequency ( Table 1).

**Table 1.** Frequency of *Kdr* alleles V1016I and F1534C in the Cayman strains across generations.

Strain	G	Details	V1016I				F1534C			
			*N*	Val/Val	Val/Ile	Ile/Ile	*N*	Phe/Phe	Phe/Cys	Cys/Cys
CAY	G1	Baseline	43	-	0.140	0.860	42	-	0.143	0.857
CAY-U	G3	unexposed	48	-	0.208	0.792	48	-	0.208	0.792
CAY-P	G3	PERM 3%	47	-	0.085	0.915	48	-	0.083	0.917
CAY-U	G6	unexposed	56	0.089	0.464	0.446	52	0.077	0.462	0.462
CAY-P	G6	PERM 3%	49	-	-	1.000	40	-	-	1.000
CAY-U	G6	1 ^st^ separation	351	0.046	0.313	0.641	*NA*	*NA*	*NA*	*NA*
CAY-U	G8	2 ^nd^ separation	*NA*	*NA*	*NA*	*NA*	416	0.269	0.510	0.221
CAY-RR	G10	screen	23	-	-	1.000	23	-	-	1.000
CAY-SS	G10	screen	23	0.978	-	0.022	23	0.978	-	0.022
CAY-SS	G0	cleaning	189	1.000	-	-	138	0.957	-	0.043

CAY-U: strain without insecticide exposure; CAY-P: strain selected with 3% permethrin; CAY-SS: homozygous for susceptible alleles without insecticide exposure; CAY-RR: homozygous for resistant alleles without insecticide exposure; CAY-RS: heterozygous by the cross between CAY-RR females and CAY-SS males; CAY-SR: heterozygous by the cross between CAY-SS females and CAY-RR males

***Kdr allele frequency.*** Both *kdr* alleles (V1016I and F1534C) showed complete linkage in all 230 individuals. The allele frequency for both *kdr* alleles in the Cayman colony was 93% at baseline with a high frequency of resistant homozygotes (86%). Selection with 3% permethrin (CAY-P) lead to *kdr* fixation within five generations. In CAY-U, *kdr* allele frequency was 68% split between a similar proportion of heterozygotes (46.4%) and resistant homozygotes (44.6%) ( Table 1).

Only 1.27% of CAY-RR died in standard WHO tube bioassays with permethrin whereas CAY-SS had a mortality rate of 82.9%.

***Resistance ratio to permethrin.*** CAY-P displayed a resistance ratio over 29x that of CAY-SS when compared to the reference colony New Orleans ( Table 2). CAY-P and the unexposed CAY-RR had similar resistance ratios of over 190x resistance compared to New Orleans. Resistance ratios in the heterozygote strains CAY-RS and CAY-SR were similar to each other, around 2x higher than CAY-SS, and 13x lower than CAY-RR.

**Table 2.** Lethal concentrations and resistance ratios of Cayman strains for permethrin.

Insecticide	Strain	LC _50_	RR _50_	LC _95_	RR _95_
Permethrin	New Orleans	0.076 (0.066–0.087)	*N/A*	0.14 (0.12–0.16)	*N/A*
	CAY-SS	0.50 (0.42–0.58)	6.58 (5.31–8.09)	0.98 (0.81–1.16)	7.00 (5.43–9.00)
	CAY-RR	15.53 (14.87–16.20)	203.31 (177.49–236.89)	25.75 (24.33–27.17)	183.18 (154.80–222.63)
	CAY-RS	1.16 (0.98–1.33)	15.16 (12.32–18.58)	2.31 (1.84–2.78)	16.43 (12.46–21.39)
	CAY-SR	1.07 (0.95–1.20)	14.01 (11.73–16.84)	1.84 (1.49–2.81)	13.06 (10.05–16.85)
	CAY-P	14.87 (14.16–15.57)	194.58 (169.59–226.99)	24.22 (22.75–25.68)	172.29 (145.31–209.70)

LC _50_: Lethal concentration for 50% mortality; RR _50_: resistance ratio for LC _50_; LC _95_: Lethal concentration for 95% mortality; RR _95_: resistance ratio for LC _95_; brackets: confidant intervals at 95%. CAY-SS: homozygous for susceptible alleles without insecticide exposure; CAY-RR: homozygous for resistant alleles without insecticide exposure; CAY-RS: heterozygous by the cross between CAY-RR females and CAY-SS males; CAY-SR: heterozygous by the cross between CAY-SS females and CAY-RR males; CAY-P: strain selected with 3% permethrin.

### Recife

**Figure 3.**
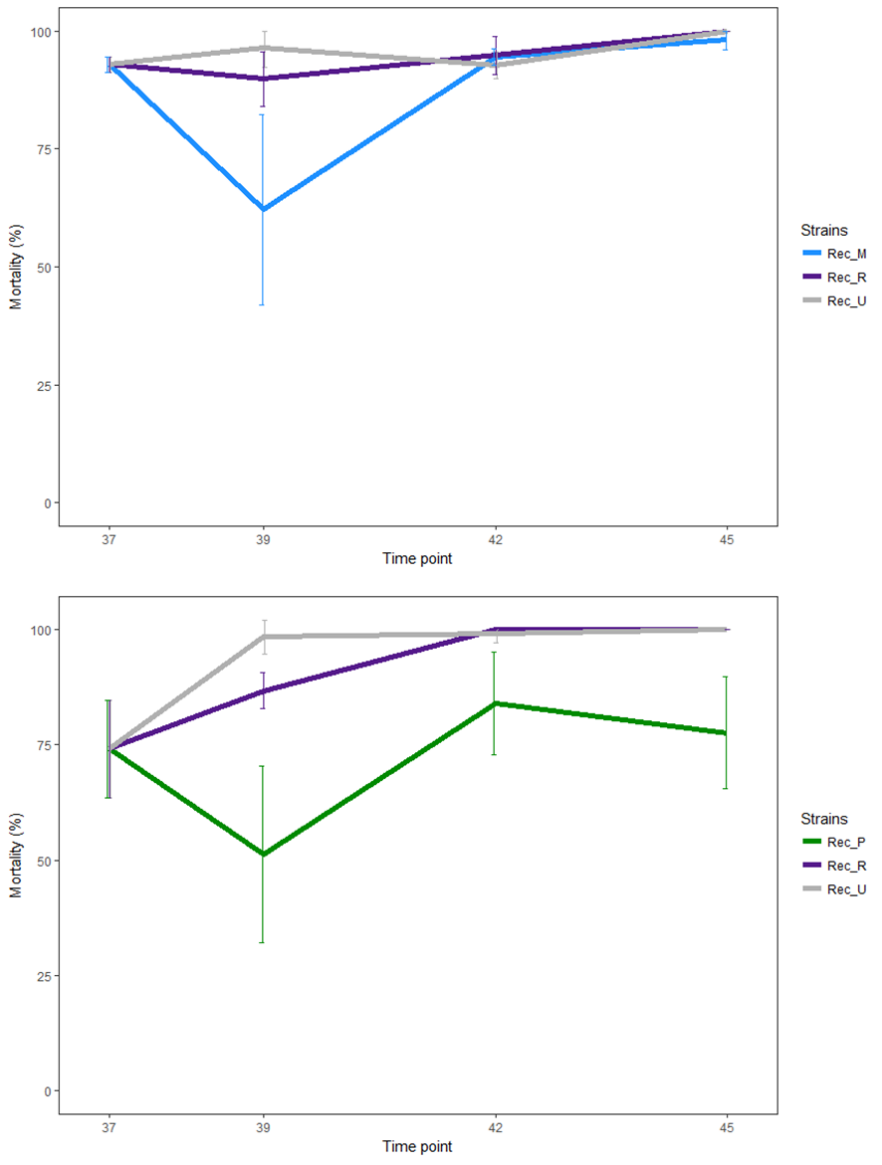
Mortality of Recife strains against WHO standard malathion 5.0% (top) and permethrin 0.75% (below). Bars represent 95% confidence intervals.

In the Recife colony, for both the malathion and permethrin-selected strains, a sharp drop in mortality to the WHO tube assay was observed, followed by a recovery then a shallower drop, rather than a gradual increase in resistance over selected generations ( Figure 3). However, the mortality of REC-P for standard WHO bioassay (0.75% permethrin) on later generations (generations 6 and 9) is around 75%, while REC-M present mortality values for standard WHO bioassay (5% malathion) above 90% on later generations ( Figure 3).

***Differential expression of detoxification genes.*** The detoxification genes with differential expression among REC strains varied between larvae and adults ( Table 3 and Table 4). In REC-R, we observed no changes in gene expression from baseline in larval stages ( Table 3), but expression of CYP9J28 and CYP6BB2 in adults increased significantly. Some genes were significantly downregulated at this stage in other Recife strains: i) REC-M (CCae3A, CYP6F3, and CYP9M9; MW: *p* <0.000666); ii) REC-U (CCae3A; MW: *p* <0.002664); iii) REC-P (CYP6F3; MW: *p* <0.002664) ( Table 3). In adults of the REC-R strain, two genes were significantly upregulated in generation G45 (CYP9J28 and CYP6BB2) (MW: *p* <0.004329). A significant upregulation in these genes was observed in REC-U (CYP9J28; MW: *p* <0.002498) and REC-M (CYP9J28 and CYP6BB2; MW: *p* <0.002165). Moreover, the CCae3A gene was upregulated in REC-M (MW: *p* <0.000250) in adults ( Table 4).

**Table 3.** Mean fold change in gene expression for larvae, Recife colony.

Strain	G	CCae3A		CYP6F3		CYP6N12		CYP9M9	
		MF	sd	MF	sd	MF	sd	MF	sd
REC-Baseline	G1	1.06	0.39	1.13	0.54	1.12	0.52	1.07	0.43
REC-U	G9	**0.34**	**0.28**	0.65	0.34	0.66	0.56	0.78	0.47
REC-R	G9	0.73	0.23	0.92	0.38	1.81	0.7	1.70	0.82
REC-M	G9	**0.38**	**0.12**	**0.41**	**0.07**	0.59	0.10	**0.20**	**0.05**
REC-P	G9	1.77	0.39	**0.41**	**0.13**	0.95	0.38	0.57	0.19

G: Generation; MF: mean fold; sd: standard deviation. REC-U: strain without insecticide exposure; REC-R: strain with temephos exposure; REC-M: strain selected for malathion; REC-P: strain selected for permethrin. Bold: Expression values significantly different from Baseline (Holm–Bonferroni method)

**Table 4.** Mean fold change in gene expression for adults, Recife colony.

Strain	G	CCae3A		CYP9J28		CYP6N12		CYP6BB2	
		MF	sd	MF	sd	MF	sd	MF	sd
REC -Baseline	G1	1.35	0.79	1.04	0.29	1.05	0.31	1.39	0.82
REC-U	G9	1.75	0.43	**2.63**	**0.47**	0.63	0.25	1.41	0.37
REC- R	G9	1.7	0.52	**2.56**	**0.31**	0.74	0.20	**3.34**	**0.75**
REC-M	G9	**2.89**	**0.37**	**3.37**	**2.11**	1.73	0.34	**3.99**	**0.90**
REC-P	G9	1.56	1.00	0.72	0.38	0.74	0.22	1.95	0.65

G: Generation; MF: mean fold; sd: standard deviation. REC-U: strain without insecticide exposure; REC-R: strain with temephos exposure; REC-M: strain selected for malathion; REC-P: strain selected for permethrin. Bold: Expression values significantly different from Baseline (Holm–Bonferroni method).

***Resistance ratio to permethrin, malathion and temephos.*** Nearly all strains were more resistant to all insecticides than New Orleans. REC-P was as much as 4x more resistant to permethrin than REC-U, and REC-R ( Table 5). REC-R and REC-M were slightly more resistant to malathion (~1.4x) than REC-U. REC-R, REC-M and REC-P were more resistant to temephos (>2.5x) than REC-U.

**Table 5.** Lethal concentrations and resistance ratios of Recife strains for three insecticides ( *i.e.* permethrin, malathion and temephos).

Insecticide	Strain	LC _50_	RR _50_	LC _95_	RR _95_
Permethrin	New Orleans	0.076 (0.066–0.087)	*N/A*	0.14 (0.12–0.15)	*N/A*
	REC-U	0.18 (0.12–0.25)	2.41 (1.58–3.34)	0.36 (0.22–0.49)	2.53 (1.53–3.68)
	REC-R	0.23 (0.17–0.29)	3.01 (2.16–3.97)	0.44 (0.32–0.56)	3.11 (2.17–4.24)
	REC-P	0.91 (0.84–0.98)	11.09 (9.52–13.09)	1.73 (1.58–1.88)	12.32 (10.26–15.23)
Malathion	New Orleans	0.51 (0.44–0.57)	*N/A*	0.98 (0.85–1.11)	*N/A*
	REC-U	0.75 (0.68–0.82)	1.48 (1.27–1.74)	1.57 (1.44–1.71)	1.60 (1.37–1.89)
	REC-R	1.18 (1.08-1.27)	2.32 (2.02-2.70)	2.22 (2.01-2.43)	2.25 (1.92-2.67¬)
	REC-M	1.23 (1.12–1.35)	2.43 (2.09–2.85)	2.65 (2.37–2.93)	2.70 (2.28–3.21)
Temephos	New Orleans	0.012 (0.012–0.013)	*N/A*	0.024 (0.022–0.026)	*N/A*
	REC-U	0.15 (0.15–0.15)	12.27 (11.52–13.12)	0.26 (0.25–0.27)	11.03 (10.14–12.04)
	REC-R	0.40 (0.39-0.42)	32.74 (30.51-35.20)	0.90 (0.86-0.94)	37.65 (34.56-41.14)
	REC-M	0.42 (0.40–0.44)	34.20 (31.81–36.83)	0.76 (0.73–0.80)	31.92 (29.30–34.88)
	REC-P	0.40 (0.38–0.41)	32.13 (29.94–34.55)	0.70 (0.66–0.73)	29.12 (26.76–31.79)

LC _50_: Lethal concentration for 50% mortality; RR _50_: resistance ratio for LC _50_; LC _95_: Lethal concentration for 95% mortality; RR _95_: resistance ratio for LC _95_; brackets: confidant intervals at 95%. REC-U: strain without insecticide exposure; REC-R: strain with temephos exposure; REC-M: strain selected for malathion; REC-P: strain selected for permethrin.

## Discussion

Inference of insecticide resistance in adults of *Ae. aegypti* is rarely performed by quantitative methods. A recent review ( Moyes *et al.*, 2017) highlights the lack of literature that calculate resistance ratios based on dose-response, lethal concentration or lethal time (see S2 file in ( Moyes *et al.*, 2017)). This practice limits comparative analysis of our results with other resistance studies. Resistance ratios for permethrin in CAY with homozygous resistance alleles (II/CC; CAY-RR and CAY-P) were of a similar magnitude to homozygous resistant *Ae. aegypti* from Cayman Islands populations ( Harris *et al.*, 2010). In other field populations, a significant positive correlation between the frequency of IICC individuals and resistance ratio for permethrin was also observed, which indicates a higher resistance for these double homozygotes ( Estep *et al.*, 2018). The lack of variation in permethrin resistance ratio between IICC strains regardless of selection pressure indicates that this allele is primarily responsible for the phenotype observed. Moreover, the other CAY strains presented lower resistance ratios to permethrin. The susceptible double homozygous strain (VV/FF; CAY-SS) had a resistance ratio similar to susceptible homozygous field populations (RR: 0.8 – 7.0) in Asia ( Brengues *et al.*, 2003) and lower than REC-P (the selected strain for permethrin in REC). This intermediate level of resistance in heterozygotes is consistent with the recessive nature of *kdr* alleles in mosquitoes and other dipterans ( Gomes *et al.*, 2017; Huang *et al.*, 2004; Saavedra-Rodriguez *et al.*, 2007). However, the combination of multiple heterozygote *kdr* alleles in *Ae. aegypti* can present a stronger resistance phenotype in the future, as is observed in Thailand where triple heterozygotes (S/P989 + V/G1016 + F/C1534) had a higher resistance ratio to deltamethrin than *kdr* homozygotes at F1534C ( Plernsub *et al.*, 2016).

Changes in temephos resistance in our REC strains differed from previous studies in two primary ways. First, the resistance level in our REC-R temephos selected strain was lower than the resistance level reported in previous studies where REC was put under similar selection pressure ( Diniz *et al.*, 2015; Melo-Santos *et al.*, 2010). This is likely because we used the New Orleans colony as the denominator in calculating the resistance ratio, while the Rockefeller colony was used in other studies. Rockefeller has a lower LC _50_ on average than New Orleans (see S1 file in ( Moyes *et al.*, 2017)). Second, in this study, temephos resistance in the REC-U unselected strain was not completely reversed while previous studies have documented reversal in a similar number of generations. This may be because our starting material had been under temephos selection pressure for longer prior to starting the experiments.

In the Recife strains, the response to the adulticides malathion and permethrin was different to the response to the larvicide temephos. REC P and REC M showed a similar LC50 to temephos as the REC-R colony and a slightly lower LC95. The gene expression within REC-R showed no significant variation compared to the baseline colony. However, REC-P, REC-M and REC-U showed downregulation of detoxification genes compared to the baseline, consistent with the lower tolerance to temephos displayed in the LC95s.

In contrast to larvicide exposure, resistance ratios for REC-P and REC-M showed a different pattern. Substantial differences between REC-P compared to either REC-R or REC-U suggest that exposure to permethrin increased the tolerance of this insecticide in the Recife colony. REC-M and REC-R present similar resistance ratios to Malathion, while the unexposed strain REC-U presents a lower resistance ratio than REC-M/REC-R. Malathion exposure over more generations will be required to increase the divergence between REC-R and REC-M phenotypes. We have experienced three main limitations in the selection of these strains: i) inconsistent blood sources, ii) time required to reverse the resistance mechanism ( Diniz *et al.*, 2015), iii) potential diversity loss associated with bottlenecks and/or high mortality due to aggressive artificial selection. We experienced difficulties in maintaining *Ae. aegypti* after a few generations using horse blood. Unfortunately, this was the only blood source available for our lab after the source of human blood was interrupted. Moreover, REC-M exhibited a drastic increase in mortality (over 90%) when we adjusted the malathion exposure concentration to 1.5% at generation 43. Mortality levels in malathion selection remained higher than 50% after this adjustment, and we had difficulties maintaining this strain post-generation 45. Future malathion selection will require a longer build-up to create a more viable resistant strain. Our method to calculate resistance ratios using WHO tubes is not able to define the individual dose received per mosquito, since mosquito weight and individual activity against the treated surface could not be measured. Topical assays, which don’t mimic natural exposure routes, do allow for controlled application and would further reduce the individual variation observed within each strain.

## Conclusions

We generated strains of *Ae. aegypti* which differ in phenotypic resistance to permethrin, malathion and temephos. The selected CAY and REC strains will allow for further research on the effects of target-site and metabolic resistance, respectively, on the life-history traits, behaviour and vector competence of this important arbovirus vector. The strains can also be used to compare the efficacy of novel insecticide formulations in strains with similar genetic backgrounds and different mechanisms of resistance.

## Data availability

### Underlying data

Open Science Framework: WT Seed project, https://doi.org/10.17605/OSF.IO/8DQ9A ( Reimer, 2020). Project registered on 10 ^th^ July 2020 (osf.io/f49jg).

This project contains the following underlying data:

- Raw values for insecticide selection mortality per generation
- Raw values for mortality in WHO bioassay
- CT values for detoxification genes in larvae
- CT values for detoxification genes in adults
- Raw values for mortality to a range of insecticide concentrations, used to calculate LC50, LC95 and resistance ratios

### Extended data

Open Science Framework: WT Seed project, https://doi.org/10.17605/OSF.IO/8DQ9A ( Reimer, 2020). Project registered on 10 ^th^ July 2020 (osf.io/f49jg).

This project contains the following extended data:

- Table S1. Insecticide selection mortality (%) for Cayman and Recife strains against standard WHO tube concentrations.
- Table S2. Primers and probes for TaqMan® SNP Genotyping Assays for Vgsc-1016 and Vgsc-1534 alleles.
- Table S3. Primers for qPCR screening of genes associated with insecticide resistance and controls.

Data are available under the terms of the Creative Commons Attribution 4.0 International license (CC-BY 4.0).
