# Supplementary material for: Insecticide resistance selection and reversal in two strains of Aedes aegypti
Source: Wellcome Open Res. 2020 Dec 16;5:183. Originally published 2020 Jul 31. [Version 2] doi: 10.12688/wellcomeopenres.15974.2 (PMC7814284; doi:10.12688/wellcomeopenres.15974.2)
Supplement: Peer review history

# Peer review: Reviewer response for version 2

**DOI:** 10.21956/wellcomeopenres.18162.r42088

**Published:** 2021-01-18

**Authors:** Qian Han

**Affiliations:** 1 Laboratory of Tropical Veterinary Medicine and Vector Biology, School of Life and Pharmaceutical Sciences, Hainan University, Haikou, China

The manuscript describes a study to develop multiple *Aedes aegypti *strains with resistance to different insecticides. The study includes two resistant colonies of *Ae. aegypti*, selection of eight strains with different profiles of resistance after exposure to different insecticides, calculation of resistance ratios for each insecticide for the selected and unselected strains, frequency of kdr alleles and some gene expression analysis. The manuscript is in an important area of methodology study understanding mechanisms underlying insecticide resistance.

 After revision based on the comments from Reviewer 1, the manuscript was well organized and written.

 Minors

1. Mosquito name typing. Keywords: *Aedes aegypti* in italic; Introduction, the second paragraph: *Aedes spp*. *Aedes *in italic, spp. in normal font; Methods: Aedes aegypti should be written in *Ae. aegypti *in italic.
2.  Subscript fonts. Discussion, the third paragraph: “LC50, LC95”, here 50 and 95 should be written in subscript. Also correct other similar errors in the sections of Method and Data availability.
3. To be consistent in typing “Cayman”: “CAYMAN”.“Cayman” or “CAY”? Same to “RECIFE”.

Is the rationale for developing the new method (or application) clearly explained?

Yes

Is the description of the method technically sound?

Yes

Are the conclusions about the method and its performance adequately supported by the findings presented in the article?

Yes

If any results are presented, are all the source data underlying the results available to ensure full reproducibility?

Yes

Are sufficient details provided to allow replication of the method development and its use by others?

Yes

Reviewer Expertise:

Vector Biology, Biochemistry and Molecular Biology.

I confirm that I have read this submission and believe that I have an appropriate level of expertise to confirm that it is of an acceptable scientific standard.

# Peer review: Reviewer response for version 2

**DOI:** 10.21956/wellcomeopenres.18162.r41855

**Published:** 2021-01-07

**Authors:** Leticia Smith

**Affiliations:** 1 National Institutes of Health, Rockville, MD, USA

The authors addressed all of my previous comments and I have no new comments on the contents of the article. There were a few typos in the "Determination of resistance ratio..." section in the Methods. Other than that, this article provides a clear description of the methods used to create these insecticide resistant mosquito strains.

Is the rationale for developing the new method (or application) clearly explained?

No

Is the description of the method technically sound?

Partly

Are the conclusions about the method and its performance adequately supported by the findings presented in the article?

Yes

If any results are presented, are all the source data underlying the results available to ensure full reproducibility?

Yes

Are sufficient details provided to allow replication of the method development and its use by others?

Partly

Reviewer Expertise:

Insecticide resistance, mosquito biology, insect physiology.

I confirm that I have read this submission and believe that I have an appropriate level of expertise to confirm that it is of an acceptable scientific standard.

# Peer review: Reviewer response for version 1

**DOI:** 10.21956/wellcomeopenres.17519.r39800

**Published:** 2020-08-17

**Authors:** Leticia Smith

**Affiliations:** 1 National Institutes of Health, Rockville, MD, USA

This study used two insecticide resistant parental colonies of *Aedes aegypti* to develop multiple strains with resistance to different insecticides. The aim was to create these strains as a tool so that the mechanisms of resistance can be studied without interference from varying genetic backgrounds. Understanding insecticide resistance in mosquitoes is as important now as ever and because insecticides will continue to be used to control disease vectors in the foreseeable future, understanding the mechanisms underlying insecticide resistance is critical for vector control. For this reason, having multiple strains with the same genetic background but different resistance mechanisms and resistance to different insecticides is a great value to the insecticide resistance research community. 

 Below are my suggestions and specific points related to the questions above that I hope will improve this publication.

 The rationale for developing the new method wasn’t clearly explained, but perhaps part of my confusion is that there didn’t seem to be anything new about the method. The rationale for developing the strains was clearly articulated, but the method of generating strains with the same genetic background through exposing versus not exposing sub-samples of the population to insecticides has been done numerous times already. Also selecting strains based on resistance genotype has been done before. I may have simply missed it, but perhaps the novelty of this method needs to be elaborated and/or more clearly articulated. As it is now, it seems like the goal was to simply select strains from a parental colony. 

 The description of the selection method was technically sound, but the evaluation of resistance part of the method could use some improvement. For the detection of *kdr* mutations, is there a reason why V410L was not checked? This is a common mutation in *Ae. aegypti* in the Americas and was only first detected after the initial study that collected the parental strain was published. How were the metabolic genes selected? Are they known to be responsible for resistance in the Recife region where the colony came from? These genes can vary across populations and by insecticide selection, so some clear explanation of how these particular ones were tested is needed. Also, why was this assay done only on the REC strains and not CAY? Some of these genes are also associated with pyrethroid resistance and are likely involved in the resistance observed in the CAY strain. Perhaps insecticide bioassays using CYP, carboxylesterase, or GST synergists would be a better evaluation of metabolic resistance than gene expression? For determining the resistance ratios, why use the WHO assay? 

 As far as details provided to allow replication of the method development and its use by others, some important information is missing or needs clarification. More information on the parental colonies would be helpful. Although the citations provide information on where the strains originated from, they do not say how many generations the strains have been in the lab prior to this study, or whether they were previously selected for insecticide resistance in the lab or are the original field collected colony. The source and stereochemistry (when applicable) for the insecticides used needs to be provided. The generation numbers used are a bit confusing. It would be helpful if only the generations from this study were used and not a mix between generations selected and what might be total number of generations in the lab. Also, in the mosquito selection regime section, the number of generations don’t seem to add up. After 5 generations without exposure, wouldn't that be generation 5? If the authors mean after five additional generations without exposure, wouldn't that be generation 15 (10 + 5)? Figure 1 shows the selection process, but that doesn’t seem to match the selection process described in the text. CAY-P was selected at every generation, not every third generation. REC-R was selected as larvae, so not using the WHO bioassay, and REC-M and REC-P seems to have been selected a total of nine generations (the figure shown only three selections). What was the calculation or method used to determine LC _50_ and LC _95_? R is a programming language and the software is only an environment to perform computations, this gives no information of how the LC _50_ was calculated. Maybe a link to a GitHub project or profile with the code used? Or a citation for the calculation methodology used? The authors also need to be more specific about how the resistance ratio was calculated. How were they compared? How was significance determined? How was control mortality accounted for? For figures 2 and 3, change “time point” to “generations” and to make it clearer and add a description of what the bars represent (error, st. dev., 95%CI?).

 The authors provide a great statement in the beginning for the discussion about the need to perform more dose-response curves in insecticide resistance studies, but sadly this study also didn’t exactly provide a dose-response curve. The issue with using a concentration, such as in the WHO assay, is that the exact amount of insecticide the individuals were exposed to remains unknown. Some animals may be more active and therefore be exposed to more by walking around the tube more than others. Also, an individual that is being affected may fall on its side more frequently and in the process get a higher exposure than a healthy animal that is standing straight. While LC assays are great for certain purposes, they are not optimal for determining resistance ratios and dose-response curves. Would it be possible to do topical assays on the final strains to get a dose response RR instead of a concentration response RR?

 In regard to using the New Orleans colony as the denominator in calculating the resistance ratio, a recent study showed that the NO strain has multiple *kdr* mutations ^1^, just something to keep in mind. In regard to the potential loss of diversity associated with bottlenecks limitations mentioned by the authors in the discussion section, perhaps backcrossing back to the parental colony after each selection could reduce this problem. With enough selections and backcrossing you should retain only the resistance mutations (if they were present in the original population) and any genes that are linked to the resistance alleles (this one is harder to separate out).

 On a very minor note, the citation for Harris *et al.* 2010 was listed twice.

Is the rationale for developing the new method (or application) clearly explained?

No

Is the description of the method technically sound?

Partly

Are the conclusions about the method and its performance adequately supported by the findings presented in the article?

Yes

If any results are presented, are all the source data underlying the results available to ensure full reproducibility?

Yes

Are sufficient details provided to allow replication of the method development and its use by others?

Partly

Reviewer Expertise:

Insecticide resistance, mosquito biology, insect physiology.

I confirm that I have read this submission and believe that I have an appropriate level of expertise to confirm that it is of an acceptable scientific standard, however I have significant reservations, as outlined above.

## References

[1] : Evidence for both sequential mutations and recombination in the evolution of kdr alleles in Aedes aegypti. *PLoS Negl Trop Dis*.14(4) : 10.1371/journal.pntd.0008154 e0008154 10.1371/journal.pntd.0008154 32302303PMC7164583

# Response

**Published:** 2020-12-02

**Authors:** Lisa Reimer

**Affiliations:** Liverpool School of Tropical Medicine, UK

Thank you to Reviewer 1 for the helpful and insightful comments. We have made additions to the manuscript to clarify the scope, re-analysed the data and provided a few additional methodological details and rationale. There were quite a few suggestions for additional research or using different methods than what we have (such as tarsal assays, synergist assays, sequencing). While these are all valid suggestions they were beyond the scope of this methods article. We have hopefully clarified the aims of this paper, which is to describe the methods underlying the selection and creation of the 8 mosquito strains. These strains have been provided to other researchers to address some of the key questions about insecticide resistance and the purpose of this article is to provide the complete background on the process. Our primary outcome was the resistance ratio (compared to a susceptible lab strain). Where we chose a few markers (kdr alleles, metabolic resistance genes, knockdown in WHO assays to select insecticides) the purpose was to track the impact the process of selection was having not to provide a complete characterisation of all possible mechanisms. We have provided a response to the below comments and have uploaded a new version of the manuscript to reflect the changes.

Comment 1

The rationale for developing the new method wasn’t clearly explained, but perhaps part of my confusion is that there didn’t seem to be anything new about the method. The rationale for developing the strains was clearly articulated, but the method of generating strains with the same genetic background through exposing versus not exposing sub-samples of the population to insecticides has been done numerous times already. Also selecting strains based on resistance genotype has been done before. I may have simply missed it, but perhaps the novelty of this method needs to be elaborated and/or more clearly articulated. As it is now, it seems like the goal was to simply select strains from a parental colony.

 This is correct, what we present here is not completely novel methodology but it is a record of the methods used to develop these mosquito resources and it provides an initial characterisation of resistance status. These strains have been shared with other researchers who are currently investigating different aspects of insecticide resistance and we wanted to have a published record of how the resources were established. While the methods are known amongst the scientific community, these selected strains are still a unique resource which allows for the study of multiple resistant phenotypes. We request editorial advice on the most appropriate classification for this manuscript.

Comment 2

The description of the selection method was technically sound, but the evaluation of resistance part of the method could use some improvement. For the detection of kdr mutations, is there a reason why V410L was not checked? This is a common mutation in Ae. aegypti in the Americas and was only first detected after the initial study that collected the parental strain was published. How were the metabolic genes selected? Are they known to be responsible for resistance in the Recife region where the colony came from? These genes can vary across populations and by insecticide selection, so some clear explanation of how these particular ones were tested is needed. Also, why was this assay done only on the REC strains and not CAY? Some of these genes are also associated with pyrethroid resistance and are likely involved in the resistance observed in the CAY strain. Perhaps insecticide bioassays using CYP, carboxylesterase, or GST synergists would be a better evaluation of metabolic resistance than gene expression? For determining the resistance ratios, why use the WHO assay?

**CAYMAN** has been maintained in laboratory since 2008 by LITE and is screened for two *kdr *alleles (F1534C and V1016I). Moyes et al. (2017) was an important reference in our rationale for the choice to continue screening F1534C and V1016I since they are considered key mutations associated with pyrethroid resistance in *Aedes aegypti *when compared with other *kdr *alleles. We understand that V410L is among the many *kdr* alleles of *Aedes aegypti* in the Americas, and was detected approximately 10 years after our colony was established (though was found in historic samples).   We chose to continue the tracking of F1534C and V1016I to perform our selection process. Our results indicate that these two resistance alleles are linked in CAYMAN and it is possible that other *kdr* alleles exist in this strain. The information about alternative resistant alleles which may be contributing further to the resistant phenotype can be explored through sequencing studies which are currently underway by other researchers. We did not screen detox genes in CAYMAN for characterization, since resistant phenotypes suggest similar resistance for both homozygotic resistant strains (CAY-P, CAY-RR).

**RECIFE**: We selected a battery of detox genes associated with metabolic resistance in *Aedes aegypti *from Americas (based on the literature) to perform an initial screen in our strains. The purpose of the initial screen was to determine whether the selection process was having an impact and to supplement the primary outcome measure which were the resistance ratios. However, as above, further research studies based on RNAseq analysis will be conducted by other researchers. We agree that transcriptomic analyses will be very valuable but they are beyond the scope of this methods article.

 We have added some information in the text to clarify genes used and the rationale:

 Line 75-81: “Here we present the resistant phenotypes after ten generations of insecticide exposure… Additionally, we present differences in a select number of resistant alleles and metabolic genes which can be used to inform further research.”

 Line 188-192: *“Four genes associated with insecticide resistance were selected to screen expression profiles in larvae (CCae3A, CYP6N12, CYP6F3 and CYP9M9: all associated with* *temephos resistance in multiple studies* *) and adults (CCae3A and CYP6N12 associated with* *temephos resistance; and* *CYP6BB2 and CYP9J28a associated with pyrethroid resistance* *in multiple studies, Moyes et al. 2017* *).”*

 We also updated the abstract to clarify which alleles were tracked during the selection.

**Bioassays with synergist:** We agree that studies with synergists and antagonists are interesting for determining metabolic resistance, but this was not a priority during our selection process.

**Quantitative approach with WHO assays: **we address this concern under comment 4 below

Comment 3

As far as details provided to allow replication of the method development and its use by others, some important information is missing or needs clarification. More information on the parental colonies would be helpful. Although the citations provide information on where the strains originated from, they do not say how many generations the strains have been in the lab prior to this study, or whether they were previously selected for insecticide resistance in the lab or are the original field collected colony. The source and stereochemistry (when applicable) for the insecticides used needs to be provided. The generation numbers used are a bit confusing. It would be helpful if only the generations from this study were used and not a mix between generations selected and what might be total number of generations in the lab.  Also, in the mosquito selection regime section, the number of generations don’t seem to add up. After 5 generations without exposure, wouldn't that be generation 5? If the authors mean after five additional generations without exposure, wouldn't that be generation 15 (10 + 5)? Figure 1 shows the selection process, but that doesn’t seem to match the selection process described in the text. CAY-P was selected at every generation, not every third generation. REC-R was selected as larvae, so not using the WHO bioassay, and REC-M and REC-P seems to have been selected a total of nine generations (the figure shown only three selections). What was the calculation or method used to determine LC50 and LC95? R is a programming language and the software is only an environment to perform computations, this gives no information of how the LC50 was calculated. Maybe a link to a GitHub project or profile with the code used? Or a citation for the calculation methodology used? The authors also need to be more specific about how the resistance ratio was calculated. How were they compared? How was significance determined? How was control mortality accounted for? For figures 2 and 3, change “time point” to “generations” and to make it clearer and add a description of what the bars represent (error, st. dev., 95%CI?).

**Colony: **We include description for all three colonies used in this study (lines 85-100).

 “A Total of three colonies from *Aedes aegypti *were used in this study:

 Colony CAYMAN (CAY) was originally established in 2008 with *Aedes aegypti *collected in Grand Cayman island (Caribbean). This colony is highly resistant to pyrethroids and DDT, attributed to *kdr* alleles (F1534C and V1016I), and has been routinely selected with 0.75 % permethrin for 1 hour in the Liverpool Insect Testing Establishment (LITE: https://lite.lstmed.ac.uk/lite-facilities/lite-insectaries/aedes-aegypti-cayman).

 Colony RECIFE (REC) was originally established in 2004 with *Aedes aegypti* collected in Araripina, Brazil (7° 32’ S and 40° 34’ W; Melo-Santos et al. 2010). This colony is resistant to temephos (OP), and biochemical assays indicate a higher activity of multiple detox enzyme families. Larvae from this colony have been routinely selected with 0.5 mg/L temephos for 24 hours.

 Colony NEW ORLEANS is a susceptible colony established at LSTM in the 1970s. This colony originated in New Orleans (USA) and it is maintained in laboratory without insecticide exposure. Routine screening for target-site mutations associated with resistance indicates a lack of *kdr *alleles in the sodium channel and resistant alleles in Ace-1 (LITE: https://lite.lstmed.ac.uk/lite-facilities/lite-insectaries/aedes-aegypti-new-orleans).”

**Stereochemistry: **We include more information about the insecticides (lines 129-132)

  “The adult mosquitoes were selected with either permethrin (Sigma Aldritch, Pestanal®, CAS # 52645-53-1, >95.0% sum of cis+trans 97.8%) or malathion (Sigma Aldritch, Pestanal®, CAS # 121-75-5) and larvae were selected with temephos (CHEM SERVICE INC., CAS # 3383-96-8) (see “ *Mosquito selection regime*”).”

**Generations: **We agree with the reviewer about the generation information in the text. To avoid confusion, we use the generation number starting in the beginning of our experiment for both Recife (G1-G10) and Cayman (G1-G11). The information about colony age at the beginning of our experiment was only included in the figure 1.

 We updated:

- The text of the manuscript (mainly methods);
- Tables 1, 3, and 4;
- Figures 1 to 3;
- supplementary information: CT values adults.csv; Mortality after insecticide selection.csv; WHO bioassay results.csv; Table S1. Insecticide selection total mortality.csv.

**Information about analysis:**

 Thank for your comment. We recognize that the information about analysis was incomplete.

- We include more details about the analysis and the R script.

Lines 235-244: “This process was repeated for each concentration in the assay. Larval mortality was recorded at 24 hours and Abbot’s formula (Abbot 1925) was used to adjust mortality when necessary. The data from bioassays was organized by concentration in each biological replicate. These data were used to create general linear models (G.L.M.) with “logit” models for each strain using “glm” function in statistical software R-2.15.2. Lethal concentration and 95% confidence intervals were calculated using the r-package “MASS”.  Resistance ratios were calculated based on comparison of LC _50_ and LC _95_ from each strain against the reference colony New Orleans, which is fully susceptible to all three insecticides. Confidence intervals for ratios were calculated using the method MOVER-R (Newcombe 2016) presented in the R-package “pairwiseCI.”

- We update the Table 2 and 5

- We update the information about resistance ratios in Results (lines 273-277, and lines 297-300).

- We also include new references:

Abbott, W.S. 1925. A method of computing the effectiveness of an insecticide. *J. Econ. Entomol.* **18**: 265–267. The Oxford University Press.

 Newcombe RG. 2016. MOVER-R confidence intervals for ratios and products of two independently estimated quantities. *Stat Methods Med Res* **25**:1774–1778, SAGE Publications

**Figure 2 and 3:**

 We include “Bars represent 95% CI.”

Comment 4

The authors provide a great statement in the beginning for the discussion about the need to perform more dose-response curves in insecticide resistance studies, but sadly this study also didn’t exactly provide a dose-response curve. The issue with using a concentration, such as in the WHO assay, is that the exact amount of insecticide the individuals were exposed to remains unknown. Some animals may be more active and therefore be exposed to more by walking around the tube more than others. Also, an individual that is being affected may fall on its side more frequently and in the process get a higher exposure than a healthy animal that is standing straight. While LC assays are great for certain purposes, they are not optimal for determining resistance ratios and dose-response curves. Would it be possible to do topical assays on the final strains to get a dose response RR instead of a concentration response RR?

 We understand the critique on using WHO assays to calculate lethal concentrations and resistance ratios. The calculation of doses based on mosquito weight may refine a resistance ratio calculation reducing certain limitations of WHO assays (or similar assays such as CDC bottle assay). However, topical assays also present disadvantages to study insecticide resistance since they require immobilization (e.g. using cold temperature, CO _2_) and insecticide is normally apply in thorax or head (not the most common area of entry in the field). For this reason, we believe that WHO assays are a valid method for this manuscript, and they are a clear improvement over qualitative assays with reference concentrations.

 We have included a discussion on the limitations of WHO assays for quantitative approach:

 Lines 359-364: “Our method to calculate resistance ratios using WHO tubes is not able to define the individual dose received per mosquito, since mosquito weight and individual activity against the treated surface could not be measured. Topical assays, which don’t mimic natural exposure routes, do allow for controlled application and would further reduce the individual variation observed within each strain.”

 We also added to the discussion:

 Lines 303-306: “Inference of insecticide resistance in adults of *Ae. aegypti* is rarely performed by quantitative methods. A recent review (Moyes *et al.*, 2017) highlights the lack of literature that calculate resistance ratios based on dose-response, lethal concentration or lethal time (see S2 file in (Moyes *et al.*, 2017)).”

Comment 5

In regard to using the New Orleans colony as the denominator in calculating the resistance ratio, a recent study showed that the NO strain has multiple kdr mutations1, just something to keep in mind.

 1. Fan Y, O'Grady P, Yoshimizu M, Ponlawat A, et al.: Evidence for both sequential mutations and recombination in the evolution of kdr alleles in Aedes aegypti. PLoS Negl Trop Dis. 14 (4): e0008154 PubMed Abstract | Publisher Full Text

 Thank you for the concern. However, our susceptible New Orleans colony is an old colony without *Kdr* alleles such as “Liverpool”, and “Rockefeller” (see comment 3). Moreover, Fan et al. is focused on recent collections from Americas and Africa that includes recent field collection from New Orleans.

 We include the description of all colonies used in the study (Lines 88-105).

In regard to the potential loss of diversity associated with bottlenecks limitations mentioned by the authors in the discussion section, perhaps backcrossing back to the parental colony after each selection could reduce this problem. With enough selections and backcrossing you should retain only the resistance mutations (if they were present in the original population) and any genes that are linked to the resistance alleles (this one is harder to separate out).

 Thank you for this suggestion.

On a very minor note, the citation for Harris et al. 2010 was listed twice.

 References were corrected to remove this duplication.
